# Simultaneous Determination of Neonicotinoid and Carbamate Pesticides in Freeze-Dried Cabbage by Modified QuEChERS and Ultra-Performance Liquid Chromatography–Tandem Mass Spectrometry

**DOI:** 10.3390/foods12040699

**Published:** 2023-02-06

**Authors:** Bingxin Yang, Sheng Wang, Wen Ma, Guanlin Li, Mengling Tu, Zhiyong Ma, Qinghe Zhang, Hongmei Li, Xianjiang Li

**Affiliations:** 1 Key Laboratory of Chemical Metrology and Applications on Nutrition and Health for State Market Regulation, Division of Metrology in Chemistry, National Institute of Metrology, Beijing 100029, China; 2 Beijing Advanced Innovation Center for Soft Matter Science and Engineering, State Key Laboratory of Organic-Inorganic Composites, College of Chemical Engineering, Beijing University of Chemical Technology, Beijing 10029, China; 3 State Key Laboratory of Natural and Biomimetic Drugs, School of Pharmaceutical Sciences, Peking University, Beijing 100191, China; 4 College of Plant Protection, Shandong Agricultural University, Tai’an 271018, China

**Keywords:** carbamate, freeze-dried cabbage, modified QuEChERS, neonicotinoid, ultra-performance liquid chromatography–tandem mass spectrometry

## Abstract

Dehydrated vegetables are popular in instant foods, but few reports have focused on their pesticide residues. This research developed and validated a modified QuEChERS method combined with ultra-performance liquid chromatography–tandem mass spectrometry to determine 19 kinds of neonicotinoid and carbamate pesticides in freeze-dried cabbage. Herein, acetonitrile/water (*v/v* = 2:1) was selected in the extraction step. Meanwhile, 4 g anhydrous magnesium sulfate and 1 g sodium chloride were applied to the partitioning step. Dispersive solid-phase extraction sorbents were selected, and liquid chromatography conditions were further optimized for dealing with the matrix effect. The limits of quantification ranged from 1.0 to 10.0 μg/kg. The validation results were acceptable, with average recoveries of 78.7–114.0% and relative standard deviations below 14.2%. The method recoveries were closely related to the volume proportion of water in the extractant. Finally, the developed method was applied to real freeze-dried cabbages and four pesticides (propamocarb, imidacloprid, acetamiprid, and thiacloprid) were detected in six samples.

## 1. Introduction

Fresh vegetables generally have a short shelf life and are not fit for long-term storage, which is a concern regarding long-term missions. Substantial studies have demonstrated that a drying process can restrain microbial growth and prolong storage time [1,2]. Among all drying processes, freeze-drying has been considered an efficient method to preserve nutrient compositions and antioxidant activities [3]. Recently, with promotion and improvement by the National Aeronautics and Space Administration, many freeze-dried (FD) ingredients have been introduced to the food market [4]. Cabbage is one of the most consumed vegetables in Asia and is very promising as a dehydrated vegetable due to its high nutrient value. Applying the freeze-drying process to cabbage has been dedicated to producing space shuttle goods, extreme-sport foodstuffs, and certified reference materials for multi-residue pesticide analysis [5,6].

In previous studies, occurrence of neonicotinoid (NEO) and carbamate (CBM) pesticides in fresh cabbage raised significant health concerns [7,8,9]. When NEOs bind to the α4β2 subtype of neuronal nicotinic acetylcholine receptors in humans, they may cause severe neurotoxicity, including tetralogy of Fallot, congenital anencephaly, autism spectrum disorder, memory loss, and finger tremor [10,11]. CBMs are reversible inhibitors of acetylcholine esterase enzymes, leading to abnormal function of nerve synapses and neuromuscular junctions, which will cause major risks to mammals. CBMs may cause impairments in the human immune system, and long-term exposure significantly increases risk of non-Hodgkin’s lymphoma [12]. Freeze-drying is carried out at a low temperature; thus, pesticide residues may be retained in FD cabbage. Additionally, a pesticide would persist for quite a long time due to the low moisture content.

Because of those adverse effects on human health, many countries and organizations have set maximum residue limits (MRLs) for NEOs and CBMs in cabbage to guarantee consumer safety. For example, the European Union (EU) established MRLs for NEOs and CBMs ranging from 0.002 to 20.0 mg/kg [13]. In China, the MRLs ranged from 0.02 to 10.0 mg/kg [14], and the United States of America had MRLs below 21.0 mg/kg [15]. See attached Table S1 for more detailed information. Until now, MRLs have not yet been established for these pesticides in FD cabbage by the above-mentioned countries and organization.

So far as we know, there is no report about NEOs and CBMs determination methods in FD cabbage. Accordingly, a robust analytical method should be established to guarantee their safety. As one of the most common sample preparation methods for pesticides, QuEChERS (quick, easy, cheap, effective, rugged, and safe) can be highlighted owing to its inherent properties, including few steps, low organic solvent consumption, suitability for multi-pesticide residue analysis, and so forth [16]. Originally developed for analyzing pesticides in fresh fruits and vegetables, QuEChERS is also applied to dry commodities, with further development in reconstitution and clean-up procedures [17,18]. For multi-pesticides analysis, the target analytes cover a wide range of polarity [19]. Thus, they tend to exhibit partitioning differences in the aqueous and acetonitrile (ACN) phases. Furthermore, due to the discrepancy between fresh and FD cabbage in terms of water content, volatile compounds, and other matrix interference [3], it is necessary to precisely optimize the sample preparation method of NEOs and CBMs in FD cabbage in the extraction and clean-up procedure.

In the past two decades, several determination techniques have been published for detection of NEOs or CBMs in food of plant origin, such as gas chromatography–mass spectrometry (GC–MS) [20], high-performance liquid chromatography-diode-array detection (HPLC-DAD) [21], and ultra-performance liquid chromatography–tandem mass spectrometry (UPLC–MS/MS/MS) [22]. In view of its short separation time [23], increased peak capacity, and excellent sensitivity [24,25], UPLC–MS/MS/MS is very attractive to detect pesticide residues in FD cabbage.

The objective of the study was to develop and validate a high-throughput, sensitive, and robust method for determining 19 kinds of NEO and CBM pesticides in FD cabbage. A modified QuEChERS method combined with UPLC–MS/MS/MS was established for this purpose. For QuEChERS, the modifications were optimized with the goal of increasing recovery and removing matrix interference. Validation and applicability of both the extraction and determination methods were also developed and finally applied to real samples. To the best of our knowledge, this was the first method for simultaneous determination of NEOs and CBMs in FD cabbage.

## 2. Materials and Methods

### 2.1. Reagents and Materials

Standards of dinotefuran (99.47% purity), nitenpyram (98.84% purity), cycloxaprid (92.70% purity), thiamethoxam (99.65% purity), clothianidin (99.12% purity), imidacloprid (98.55% purity), acetamiprid (99.78% purity), imidaclothiz (97.46% purity), and thiacloprid (99.68% purity) were obtained from Dr. Ehrenstorfer (Augsburg, Germany). Standards of propamocarb (99.30% purity), oxamyl (99.40% purity), pirimicarb (99.50% purity), aldicarb (97.00% purity), metolcarb (99.80% purity), propoxur (99.50% purity), carbofuran (99.70% purity), carbaryl (99.80% purity), isoprocarb (99.80% purity), and promecarb (99.80% purity) were obtained from Alta scientific Co., Ltd. (Tianjin, China). Chemical structures of the target NEO and CBM pesticides were exhibited in Figure S1.

ACN of HPLC grade was purchased from Merck KGaA (Darmstadt, Germany). For sample extraction, anhydrous sodium sulfate (Na_2_SO_4_) was purchased from FUCHEN Chemical Reagents (Tianjin, China); sodium chloride (NaCl) was purchased from Sinopharm Chemical Reagent Co., Ltd. (Shanghai, China). The QuEChERS CEN version extraction tube (containing 4 g of anhydrous magnesium sulfate (MgSO_4_), 1 g NaCl, 1.0 g trisodium citrate dihydrate, and 0.5 g disodium hydrogen citrate sesquihydrate), AOAC version extraction tube (containing 6 g anhydrous MgSO_4_ and 1.5 g sodium acetate), and original version extraction tube (containing 4 g anhydrous MgSO_4_ and 1.0 g NaCl) were purchased from Waters Technologies (Milford, MA, USA). HPLC grade acetic acid was obtained from Innochem Company (Beijing, China). For sample purification, the QuEChERS dispersive kit (anhydrous MgSO_4_^+^ primary secondary amine (PSA)) was purchased from Agilent Technologies (Folsom, CA, USA); graphitized carbon black (GCB) was purchased from Agilent Technologies (Folsom, CA, USA). Carboxylated multi-walled carbon nanotubes (c-MWCNTs, 5–15 nm in outer diameter, 10–30 μm in length, 3.86% carboxyl content) and MWCNTs (5–15 nm in outer diameter, 0.5–2 μm in length) were purchased from Xianfeng Technology Co., Ltd. (Nanjing, China). For the mobile phase, LC–MS grade formic acid and ammonium formate were purchased from Honeywell (Shanghai, China). Purified water was purchased from Wahaha Group Co., Ltd. (Hebei, China). For the syringe filters, hydrophilic polytetrafluoroethylene (PTFE) with a pore size of 0.22 μm was manufactured by ANPEL Laboratory Technologies (Shanghai, China).

### 2.2. Pesticide-Free FD Cabbage Preparation

About 20 kg of organic-certified cabbages was purchased from Beicaiyuan Agriculture Technology Co., Ltd. (Beijing, China). After removing the roots and decomposed leaves, the samples were chopped into strips, followed by further homogenization with a food processor (VAKNOA QUALITAT, Germany) at room temperature for 5 min. Thereafter, the cabbage paste was collected and placed into a freeze dryer (VirTis Genesis/35 L Genesis Super XL-70, USA). The temperature and vacuum pressure in the drying chamber were set at −25 °C and 200 mTorr, respectively. The process took 48 h, with the stainless-steel plate temperature gradually raised to 25 °C. Finally, the pesticide-free FD cabbage samples were ground using a medicine pulverizer (Xulang, Guangzhou, China) and stored in a deep freezer at −80 °C until use.

### 2.3. Sample Fortification

Pesticide-free FD cabbage was collected for method validation. After homogenization, blank sample was fortified with the standard solution at the concentrations of 1.0, 10.0, 100.0, and 500.0 μg/kg. Then, the samples were allowed to equilibrate for over 30 min prior to further pretreatment.

### 2.4. Real Sample Analysis

Seven FD cabbage samples were obtained from the market in Jiangsu, and each sample was ground as before. The FD cabbage was prepared with a modified QuEChERS method as follows: first, the sample was taken out from the refrigerator and restored to room temperature; 1.0 g sample was weighed into a 50 mL centrifuge tube. Afterwards, 5 mL purified water and 10 mL ACN were added for liquid phase extraction, and the mixture was agitated with multi-tube vortex mixer (Lumiere., Beijing, China) for 3 min at 2500 rpm; for phase separation, 4 g of anhydrous MgSO_4_ and 1 g NaCl were added to the tube and agitated with multi-tube vortex mixer for 1 min at 2500 rpm; the tube was then centrifuged for 5 min at 8000 rpm (3802 g) at 4 °C; for the dispersive solid-phase extraction (d-SPE) that followed, an aliquot of 2 mL of supernatant organic layer was collected into the purification tube, which contained 300 mg anhydrous MgSO_4_, 50 mg PSA, and 20 mg GCB. The tube was agitated with multi-tube vortex mixer for 3 min at 2500 rpm and centrifuged for 5 min at 8000 rpm (3802 g) at 4 °C; finally, the upper layer (0.5 mL) was collected and mixed with 1 mL mobile phase A before filtration with PTFE membrane filter and transferred for UPLC–MS/MS/MS analysis.

### 2.5. Instrument and Apparatus

A UPLC–MS/MS/MS (Waters Technologies) was used for the instrumental analysis of the extract. ACQUITY UPLC^®^ was used for LC separation and coupled to a triple quadrupole MS (TQ-S, Manchester, UK) equipped with orthogonal Z-spray electrospray ionization (ESI) interface. MassLynx 4.1 software with QuanLynx program was used for data acquisition and analysis. For chromatographic separation, an HSS T_3_ column (1.8 μm particle size; 2.1 mm × 100 mm, Waters) was utilized, and the column temperature was maintained at 30 °C. The mobile phases were composed of a mixture of 0.1% formic acid, 5 mM ammonium formate in water (phase A), and ACN (phase B), and the flow rate was set at 0.3 mL/min. For the gradient elution, the initial eluent composition was 10% phase B, and phase B reached 50% at 3 min, progressively increasing to 80% at 5 min. Finally, it was lowered to 10% at 6 min and maintained for another 2 min for equilibration. The total time of a single run was 8.0 min, and 4.5 μL of the sample was injected for quantification.

The MS/MS was operated in positive ionization mode, and data were acquired in the multiple reaction monitoring (MRM) mode with two transitions per pesticide. The transition with the highest intensity was utilized for quantitation and the other was utilized for confirmation. Capillary voltage and source offset voltage were 3.0 kV and 55 V, respectively; desolvation and source temperatures were 500 and 150 °C, respectively; cone and desolvation N_2_ gas flows were 150 and 1000 L/h, respectively. Table 1 listed the retention time (R_t_), cone voltage (CV), precursor ion, product ion, and collision energy (CE) for the 19 kinds of pesticides in the UPLC–MS/MS/MS method.

### 2.6. Method Validation

Validation of the developed method was carried out according to the SANTE/11312/2021 guidance document for determination of pesticide residues [26]. The validation included selectivity, limit of quantification (LOQ), limit of detection (LOD), linearity, matrix effect (ME), recovery, repeatability, and stability of the final extract. Blank extract from pesticide-free FD cabbage (prepared in 2.2) was used to evaluate method selectivity. Matrix-matched standard solutions were used for determining LOD, LOQ, and linearity. LOQ was determined as the concentration of signal-to-noise ratios larger than 10; LOD was defined as the lowest detectable concentration that was determined as the concentration of signal-to-noise ratios larger than 3. According to the prescription of SANTE/11312/2021, the linear range consisted of a minimum of five calibration points for each pesticide. MEs were calculated by flowing Equation (1): a positive value indicated matrix enhancement effects, while a negative value indicated the opposite. In addition, MEs were considered significant if they exceeded ±20%.

MEs = (slope of matrix –matched standard/slope of solvent standard − 1) × 100%(1)

Recovery experiments were performed in five replicates at four spiked fortification levels of 1.0, 10.0, 100.0, and 500.0 μg/kg (taking the LOQ, 10 times LOQ, or the MRLs for each pesticide as reference values). Repeatability was termed as the relative standard deviation (RSD) calculated with samples prepared and analyzed in one day or over several days. The final extract stability was evaluated at 100 μg/kg after (1) storage at room temperature in the autosampler tray for 24 h; (2) storage in a refrigerator (4 °C for three days and seven days, respectively).

### 2.7. Post-Column Infusion

Post-column infusion system could directly reflect MEs occurring during a whole chromatographic run [27]. In our study, two sets of experiments were performed with pure ACN as a “reference” and the blank sample extract. Meanwhile, a pesticide infusion mixture (100 μg/kg) was permanently infused to the LC effluent after column with a constant flow (10 μL/min) via a T-piece in Intellistart^TM^ system. The intensity of each MRM transition was recorded for every 0.36 s; thus, 1383 data points were acquired during the total run time for each pesticide. By calculations according to Equation (2), the matrix profile was obtained from the two infusion profiles.

ME_i_ = [SI_i_ (sample extract)/SI_i_ (reference) − 1] × 100% (2)

Here, SI represented the signal intensity.

If no MEs occurred, the two infusion profiles were identical and MEs were close to zero. If matrix interference influenced the ionization efficiency of target analytes, the infusion profiles of the sample extract would differ from the reference.

## 3. Results and Discussion

### 3.1. Optimization of the QuEChERS Method

QuEChERS is a simple yet flexible method with many commercial reagents and sorbents for extraction and clean-up. To achieve accurate results, the QuEChERS parameters were optimized with the goal of increasing recovery, lowering RSD, and minimizing ME. The initial conditions used to perform the optimization were as follows: 1.0 g FD cabbage sample with a spiking level of 100 μg/kg, the extraction solvent volume was 20 mL (10 mL purified water and 10 mL ACN), and the extraction time was 3 min. The phase partitioning salts were from CEN version, and the d-SPE sorbents contained 300 mg anhydrous MgSO_4_, 50 mg PSA, and 20 mg GCB.

#### 3.1.1. Sample Extraction

**Figure 1:**
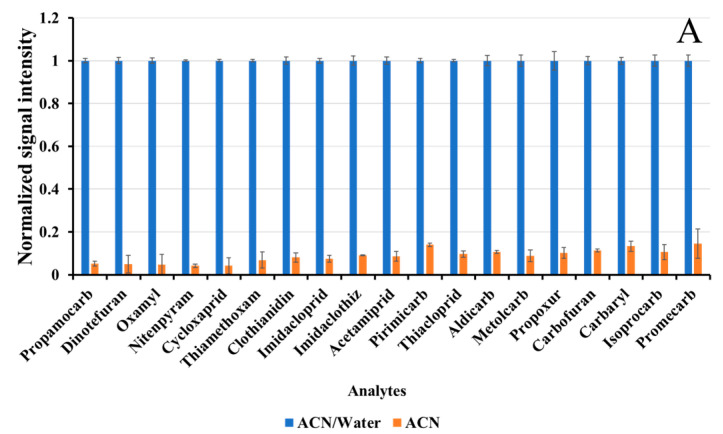

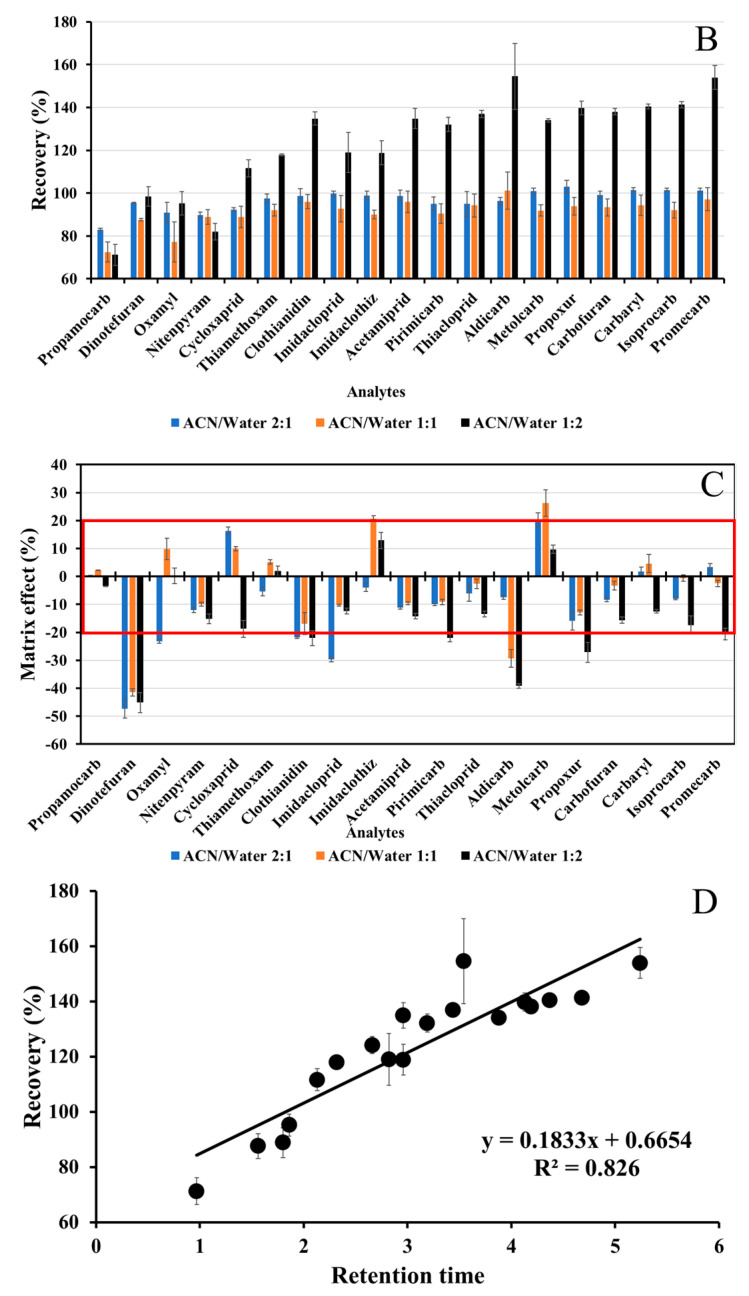
Effect of extraction parameters: (**A**) extraction efficiencies in different extraction solvents; (**B**) recoveries and (**C**) MEs in different volume proportions of ACN/water (the MEs in the red wireframe were negligible); (**D**) linear correlation between Rt and recoveries (*v*/*v* (ACN/water) = 1:2).

For FD cabbage, addition of water was recommended, which swelled the pores and allowed the ACN access into sample tissue [28]. Figure 1A showed that an ACN/water mixture provided consistently higher extraction efficiency for all analytes, while extraction with ACN was only 4.2–14.6% of the former performance. Mastovska et al. found that use of ACN/water for single extraction showed comparable extraction efficiency with ACN extraction after water reconstitution [29]. Therefore, an ACN/water mixture was chosen for FD cabbage.

Moreover, recoveries of analytes may decrease and co-extract interference may increase with different water content in the extraction solvent [30,31]. Therefore, the volume of ACN was maintained at 10 mL while the volume proportion of ACN/water was further compared (*v/v* = 2:1, 1:1, 1:2, respectively). The experimental results shown in Figure 1B,C indicated that acceptable results were obtained in the range of 2:1–1:1 (*v/v*) ACN/water since there was no significant difference in recoveries and MEs. However, it was noteworthy that two considerable biases in recoveries were found for analytes when the proportion of ACN/water was 1:2 (*v/v*). In detail, negative bias for nitenpyram and propamocarb was mainly due to their hydrophilicity, while positive bias was found for relatively hydrophobic analytes (aldicarb, metolcarb, propoxur, carbofuran, carbaryl, isoprocarb, and promecarb). As a result, the recoveries of the target analytes in the ACN/water mixture (*v/v* = 1:2) exhibited a strong linear correlation with the R_t_ (R^2^ = 0.826 in Figure 1D). The results indicated that an aqueous phase beyond the appropriate range would pry the “levers” of consistency in analyte recoveries. Finally, the ACN/water mixture (*v/v* = 2:1) was selected as the appropriate extraction solvent.

Inadequate extraction may lead to low recovery; then, it is imperative to optimize extraction time [32]. Thus, equilibrium extraction time was investigated by increasing the time from 1 min to 10 min. As shown in Figures S2 and S3, there was no significant difference in recoveries and MEs for most analytes, but the recoveries for four NEOs (nitenpyram, dinotefuran, cycloxaprid, and thiamethoxam) were moderately increased with the extraction time increasing from 1 min to 3 min. Therefore, 3 min was selected as the extraction time. The results indicated that extraction of target analytes in FD cabbage mainly depended on the volume proportion between the ACN and aqueous phases and extraction time was not a very critical factor.

#### 3.1.2. Liquid–Liquid Phase Partitioning

**Figure 2:**
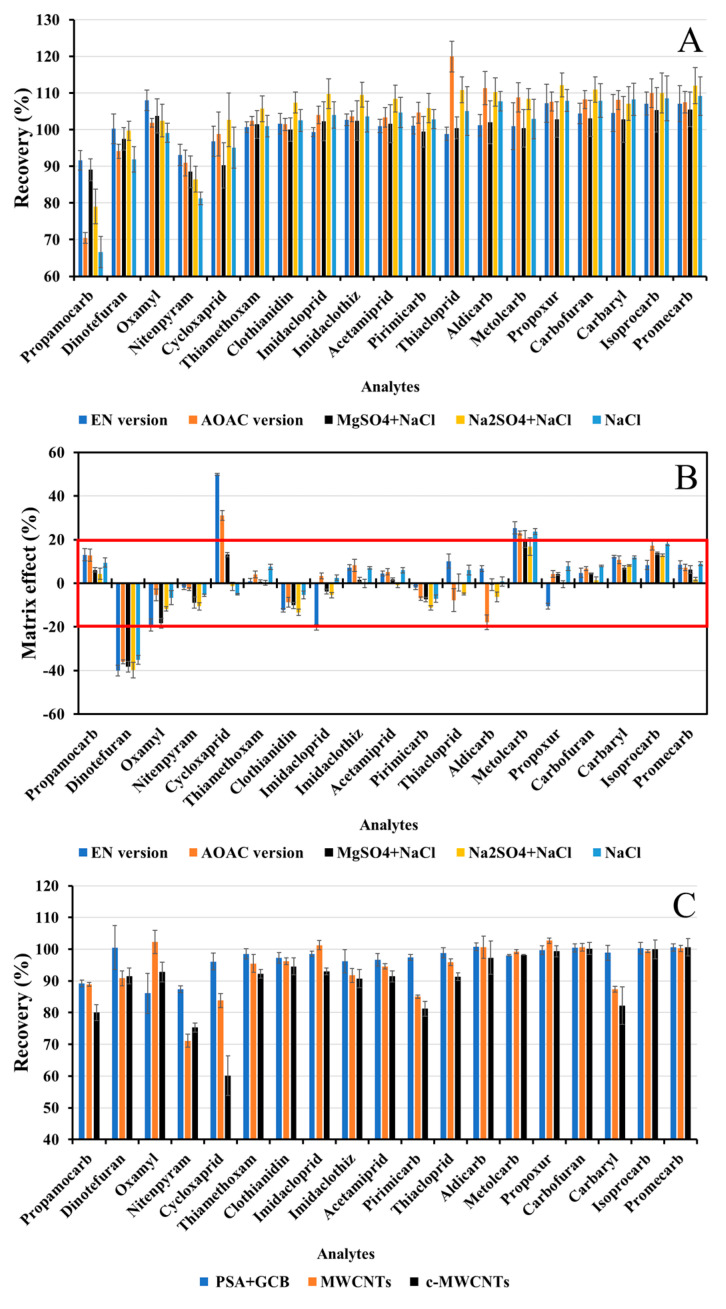

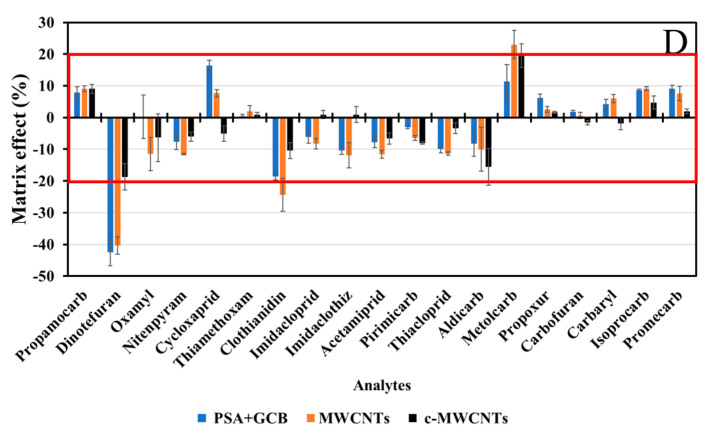
Effect of phase partitioning salts on (**A**) recoveries and (**B**) MEs; effect of d-SPE sorbents on (**C**) recoveries and (**D**) MEs (the MEs in the red wireframe were negligible).

After sample extraction, the ACN extract needed to be separated from the sample. This liquid–liquid phase partitioning was accomplished by adding partitioning salts. The partitioning salts not only control the polarity difference between the ACN and aqueous phases but also influence the pH of the sample environment, thus strongly influencing recoveries of target analytes [31]. Herein, the experiment was evaluated with two buffered partitioning salts (CEN version and AOAC version) and three unbuffered partitioning salts (original version, 4 g anhydrous Na_2_SO_4_^+^ 1 g NaCl, 3 g NaCl, respectively). As shown in Figure 2A, anhydrous MgSO_4_ provided higher recoveries for propamocarb and nitenpyram, indicating that the recoveries of relative polar analytes primarily correlate with the water content in the ACN phase. In addition, the lower solubility of anhydrous Na_2_SO_4_ in water limited its water binding capacity. Thus, anhydrous MgSO_4_ was selected as a component in partitioning salts to bind water from the sample.

Meanwhile, by evaluating MEs and matrix profiles, CEN version buffer salts had significant matrix interference on cycloxaprid and imidacloprid, as shown in Figure 2B, Figures S4 and S5. Judging from these results, the experiment demonstrated that there was no need to add buffer salts. Among them, the original version salts provided the best results in terms of lower MEs and consistently good recoveries.

#### 3.1.3. Sample Clean-Up

In this study, the initiative was to keep the clean-up procedure as simple as possible, acquire consistently good recovery, and remove serious matrix interference simultaneously. Our previous study found that the combination of PSA and GCB has commendable clean-up efficiency for co-extracts in fresh cabbage [7]. MWCNTs and c-MWCNTs also demonstrated good clean-up performance for pesticides in cucumber, apple, and orange [33]. As shown in Figure 2C,D, when FD cabbage extracts were cleaned with c-MWCNTs, the MEs for dinotefuran, cycloxaprid, and clothianidin were decreased by 23.8%, 11.4%, and 8.2%, respectively. However, non-specific adsorption also occurred and the recovery for cycloxaprid fell to 60.2%. Compared with CBMs (except for carbaryl), pharmacophores (–NO_2_ and –CN) in nine NEOs were electronegative, and carboxyl groups in the c-MWCNTs could form strong hydrogen bond interaction and increased the additional adsorption to NEOs. This was why PSA and GCB turned out to be generally applicable d-SPE sorbents for FD cabbage. Then, further optimization of the sample clean-up was not conducted; the study attempted to find better LC separation conditions to separate dinotefuran from co-eluting matrix interference in the following.

### 3.2. Optimization of UPLC–MS/MS/MS Conditions

**Figure 3:**
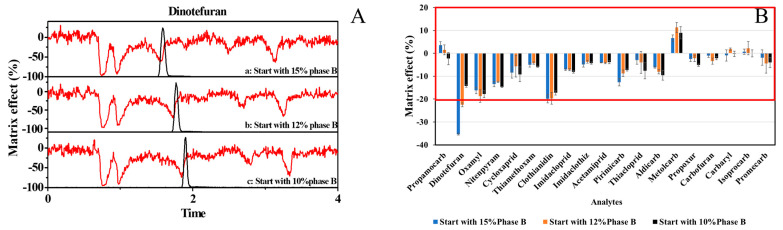
(**A**) Matrix profiles in different type LC methods from a post-column infusion of dinotefuran (a, b, c represented the gradient of the LC method); (**B**) MEs in different type LC method (the MEs in the red wireframe were negligible).

In order to find better LC separation conditions to eliminate the ME of dinotefuran, the post-column infusion system was adopted. In our former work [7], the LC elution started with 15% phase B, reached 50% phase B within 3 min, 80% phase B in 5 min, and then dropped to the initial state within 1 min. Under this condition, most target analytes were well separated from each other. However, Figure 3A(a) showed that the signal of dinotefuran was significantly suppressed by the co-eluting matrix. After optimizing LC elution conditions, the proportion of phase B in the initial mobile phase decreased from 15% to 10%. Figure 3A(b,c) showed ME profiles of blank FD cabbage samples with optimized LC conditions. The results demonstrated that dinotefuran gradually separated from the matrix-suppressing zone as the proportion of phase B decreased. Therefore, the ME of dinotefuran in FD cabbage was eliminated with the optimized LC conditions and has no significant influence on other analytes (Figure 3B).

To improve the signal intensity of target analytes, mobile phase modifiers, such as ammonium acetate and formic acid in purified water, were discussed. Table S2 showed that adding ammonium formate and formic acid resulted in a 1.2- to 12.2-fold higher response than individually using formic acid for most analytes, except for oxamyl and aldicarb. The reasons why mobile phase modifiers influenced protonation of the target analytes may be ascribed to the following theory. Proton transfer was a fundamental chemical reaction occurring in both solution and the gas phase (Equations (3) and (4)) [34,35]. Adding a small mount of formic acid and ammonium formate can push reaction 3 and 4 and promote protonation of the analytes. Interestingly, oxamyl and aldicarb did not form a stable protonated molecule [M + H]^+^ but formed a very stable sodiated ion [M + Na]^+^ in formic-acid-containing ACN solution. Consequently, the observed suppression of Na adduct ions by ammonium formate was likely due to competition between Equations (4) and (5) in the gas phase [36]. Moreover, Figure S6 showed that adding extra ammonium formate produced a symmetric peak shape compared with adding only formic acid for nitenpyram. Eventually, purified water spiked with 0.1% formic acid and 5 mM ammonium formate was selected as phase A.

M (l) + H_3_O^+^ (l) → [M + H]^+^ (l) + H_2_O (l) (3)

M (g) + NH_4_^+^ (g) → [M + H]^+^ (g) + NH_3_ (g) (4)

ACN•Na^+^ (g) + M (g) → [M + Na]^+^ (g) + ACN (g) (5)

MS/MS conditions were optimized for unambiguous identification and accurate quantification of 19 kinds of NEO and CBM pesticides at trace levels. The MS parameters were optimized by directly injecting the standard solution. One precursor ion and the two most abundant product ions were selected for each compound as confirmation ions. Detailed parameters were shown in Table 1.

In preliminary experiments, a 1.5 μL injection volume was used to test the mobile phase composition, but a high proportion of organic solvent was apt to produce obvious solvent effect for propamocarb. The most common approach to prevent solvent effect is solvent replacement with the initial mobile phase. However, the concentration process led to instability of nitenpyram and quantitative errors. Therefore, directly diluting the final extract with phase A was attempted. Figure S7 demonstrated that solvent effects could be significantly lowered with two times dilution with phase A. Hence, two times dilution with phase A was selected and the injection volume was changed to 4.5 μL accordingly. QuEChERS is a simple yet flexible method, with many commercial reagents and sorbents for extraction and clean-up. To achieve accurate results, the QuEChERS parameters were optimized with the goal of increasing recovery, lowering RSD, and minimizing ME. The initial conditions used to perform the optimization were as follows: 1.0 g FD cabbage sample with a spiking level of 100 μg/kg, the extraction solvent volume was 20 mL (10 mL purified water and 10 mL ACN), and the extraction time was 3 min; the phase partitioning salts were from CEN version and d-SPE sorbents contained 300 mg anhydrous MgSO_4_, 50 mg PSA, and 20 mg GCB.

### 3.3. Method Validation

#### 3.3.1. LODs, LOQs, and Linearity

The LODs, LOQs, and linearity were experimentally determined by fortification of pesticide-free FD cabbage samples. Table 2 presented the LODs and LOQs for the NEO and CBM pesticides. Low LODs and LOQs were achieved in the range of 0.2 to 4.0 μg/kg and 1.0 to 10.0 μg/kg, respectively. The linearity of the method was evaluated by linear regression analysis of matrix-matched calibration curves. All calibration curves were constructed at the fortification levels of 1.0, 2.0, 5.0, 10.0, 50.0, 100.0, 200.0, 500.0, and 1000 μg/kg in pesticide-free FD cabbage samples. Table 2 showed that the method had good linearity, with satisfactory correlation coefficients (R^2^ > 0.9990) for all target analytes.

#### 3.3.2.Assay Selectivity

Pesticide-free FD cabbage samples were analyzed to evaluate the selectivity of the established method. No interfering endogenous peaks appeared at the R_t_ of the target analytes. The extracted MRM ion chromatograms of the FD sample with fortified NEOs and CBMs mixture at LOQ concentration levels were shown in Figure S8. The chromatogram demonstrated the selectivity and good chromatographic characteristics of the proposed method at a low fortification level.

#### 3.3.3. Matrix Effect

Some endogenous components in the FD cabbage might be extracted to the final extract, thereby interfering with ionization of target analytes. Then, this interference would be detrimental to maintenance of the UPLC–MS/MS/MS and affect quantification of the analytical method. According to the SANTE/11312/2021 guidelines, the MEs are considered significant if they exceed ±20%. The results were shown in Table 2. Thus, with values ranging from −16.5% to 16.1%, the MEs were not significant for all analytes. To further diminish MEs, the matrix-matched standard curves were applied to compensate the matrix interference and signal irreproducibility.

#### 3.3.4. Recovery and Repeatability

For determination of recovery and repeatability, two levels of fortification must be considered: (i) a sample fortified with LOQs concentration for each analyte (*n* = 5); (ii) a sample fortified with 10 times LOQs or MRLs (set or proposed) level. Considering the difference in LOQs of target analytes, the method was validated in five replicates at four fortification concentrations: 1.0, 10.0, 100.0, and 500.0 μg/kg. Table 2 showed that the average recoveries ranged from 78.7% to 114.0%, with intraday RSD (RSD_r_) values ≤7.8%. Interday RSD (RSD_R_) values were slightly higher (≤14.2%) but lower than 15%, which was an acceptable level.

#### 3.3.5. Stability of Final Extracts

A homogenized blank extract was separated into two aliquots to investigate the stability of the target analytes. Each aliquot was fortified at 100 μg/kg for each pesticide and prepared as in Section 2.4. One aliquot was analyzed at room temperature after one day, and the other was stored at 4 °C and analyzed three and seven days later. Figures S9 and S10 showed that no significant degradation (within the acceptable range of 70–120% [26]) was observed for all analytes in the final extracts for one-day room temperature storage and three-day 4 °C storage, but, evidently, degradation occurred for oxamyl, cycloxaprid, and aldicarb for seven-day 4 °C storage. In summary, the final extract should not be stored for more than three days.

### 3.4. Real Sample Analysis

In our research, seven batches of FD cabbage samples collected from the market in Jiangsu Province were used to validate the reliability of the developed method. The results were shown in Table 3. Three NEO and one CBM pesticides were detected in six FD cabbage samples. Among them, thiacloprid and propamocarb were the most frequently detected pesticides. In contrast, imidacloprid and acetamiprid were detected in one sample. In China, carbofuran and aldicarb are banned in fruits and vegetables (including cabbage) [37]. Table S3 summarized the publication of the analytical method and the detection frequency of tested pesticides in cabbage [7,8,38,39,40]. From the above results, the detection rate of NEOs was usually higher than CBMs. This may be ascribed to the heavy use of NEOs in agriculture.

It should be remarked that MRLs for NEOs and CBMs have not been established in FD cabbage by the EU, China, and the United States. Therefore, the MRLs for fresh cabbage were taken as a reference. In such a case, the imidacloprid residue reached 78.6 μg/kg in a sample, which was above the recommended MRL in the EU (10.0 μg/kg in fresh cabbage). This result demonstrated the practicability of the method in real sample analysis, which showed great potential in daily food safety monitoring.

## 4. Conclusions

A robust and sensitive method was first established for simultaneous determination of pesticides in FD cabbage. The combination of modified QuEChERS and UPLC–MS/MS/MS provided high sample throughput to yield acceptable quantitative results for NEO and CBM pesticides. In this study, ACN/water (*v/v* = 2:1) was chosen as the extraction solvent, followed by anhydrous MgSO_4_ and NaCl (4 g + 1 g) as partitioning salt. Moreover, post-column infusion was applied to reflect the matrix profiles of optimized LC conditions. Ultimately, the ME of dinotefuran was eliminated with an initial mobile phase of 10% ACN.

Furthermore, the reliability, robustness, and practicability of the method were verified according to the SANTE/11312/2021 document. Recoveries were achieved for all target analytes from 78.7% to 114.0%, with RSDs below 14.2% and MEs ranging from −16.5% to 16.1%. For the real samples, propamocarb, imidacloprid, acetamiprid, and thiacloprid were detected in six samples, which proved that FD cabbage also contains pesticide residues. This study can also be a practical reference for determining NEO and CBM pesticides in other dehydrated vegetables.

## Figures and Tables

**Table 1:** Parameters of MS/MS for NEO and CBM pesticides.

Analytes	R_t_^(a)^ (min)	CV ^(b)^ (V)	Precursor Ion (m/z)	Product Ion 1 (m/z)	CE ^(c)^-1 (V)	Product Ion 2 (m/z)	CE-2 (V)
Propamocarb	1.71	35	189.0	102.0	17	144.0	10
Dinotefuran	1.85	30	203.0	129.0	10	113.3	10
Oxamyl	2.15	65	242.0	72.0	10	121.0	10
Nitenpyram	2.18	30	271.0	56.1	24	126.0	32
Cycloxaprid	2.56	30	323.0	125.8	36	150.9	22
Thiamethoxam	2.58	25	291.9	210.9	12	132.0	22
Clothianidin	2.89	25	250.1	168.9	12	132.0	14
Imidacloprid	3.04	35	255.9	175.0	20	209.0	20
Imidaclothiz	3.17	30	262.0	180.8	15	122.0	26
Acetamiprid	3.17	45	223.0	125.9	18	55.9	12
Pirimicarb	3.36	40	239.0	72.0	20	182.0	16
Thiacloprid	3.58	45	253.0	125.9	20	217.0	12
Aldicarb	3.69	35	213.0	89.0	16	116.0	12
Metolcarb	3.96	25	164.0	109.0	12	94.0	26
Propoxur	4.21	5	210.0	111.0	15	168.0	5
Carbofuran	4.27	30	222.0	123.0	22	165.0	12
Carbaryl	4.43	25	202.0	127.0	25	145.0	10
Isoprocarb	4.72	30	194.0	95.0	15	137.0	9
Promecarb	5.25	30	208.0	109.0	17	151.0	9

^(a)^ retention time;**^(^**^b)^ cone voltage; ^(c)^ collision energy.

**Table 2:** Linear range, coefficients (R^2^), LODs, LOQs, MEs, average recoveries, RSD_r_ (*n =* 5), and RSD_R_ (*n =* 3) of NEO and CBM pesticides.

Analytes	Linear Range (μg/kg)	Regression Equation	R^2^	LOD (μg/kg)	LOQ (μg/kg)	ME (%)	Fortification (μg/kg)	Recovery (%)	RSD_r_ (%)	RSD_R_ (%)
		y = 11,641x – 21,927					1.0	92.2	6.9	1.8
Propamocarb	1.0–500.0		0.9998	0.2	1.0	−3.4	10.0	88.7	4.5	7.0
							100.0	86.3	1.7	2.2
		y = 1026.8x − 258.23					10.0	97.9	5.7	3.7
Dinotefuran	10.0–500.0		0.9994	4.0	10.0	−14.2	100.0	97.7	1.5	2.1
							500.0	96.7	1.1	1.9
Oxamyl	10.0–500.0	y = 423.49x − 1011.8				−14.8	10.0	104.0	7.8	8.3
Oxamyl	10.0–500.0		0.9997	4.0	10.0	−14.8	100.0	98.6	3.5	2.6
							500.0	97.2	1.3	3.9
		y = 1710.8x − 3614.8					10.0	83.7	6.8	11.2
Nitenpyram	5.0–500.0		0.9997	2.0	5.0	−3.3	100.0	86.3	1.6	3.1
							500.0	85.4	1.4	3.6
		y = 31,584x + 25,868					1.0	78.7	4.9	5.3
Cycloxaprid	1.0–100.0		0.9993	0.2	1.0	16.1	10.0	81.6	3.9	5.9
							100.0	82.1	1.0	5.3
		y = 3048.3x + 21,677					1.0	99.4	7.1	4.8
Thiamethoxam	1.0–500.0		0.9998	0.2	1.0	2.8	10.0	98.2	3.3	1.9
							100.0	99.1	1.5	1.2
		y = 787.1x + 1156.7					10.0	93.8	7.3	6.3
Clothianidin	1.0–500.0		0.9998	4.0	10.0	−16.5	100.0	96.3	2.4	4.0
							500.0	97.8	2.9	2.7
		y = 1655.5x + 2906.1					1.0	97.7	4.0	8.6
Imidacloprid	1.0–500.0		0.9990	0.3	1.0	4.8	10.0	97.4	3.5	3.9
							100.0	97.9	0.9	2.2
		y = 1554.4x −4607.2					10.0	104.4	0.4	4.1
Imidaclothiz	10.0–500.0		0.9995	3.0	10.0	9.5	100.0	99.0	2.7	3.0
							500.0	98.7	0.9	2.4
		y = 15,870x + 17,493					1.0	92.1	4.0	3.0
Acetamiprid	1.0–200.0		1.0000	0.3	1.0	6.2	10.0	99.1	1.7	2.4
							100.0	99.6	0.8	0.9
Pirimicarb	1.0–500.0	y = 16,692x + 30,784	0.9998		1.0	−0.6	1.0	93.4	0.4	8.8
	1.0–500.0		0.9998	0.3	1.0	−0.6	10.0	98.7	1.2	0.7
							100.0	97.1	1.3	1.4
ThiaclopridTHI		y = 22,576x + 21,938					1.0	99.8	1.7	1.0
	1.0–200.0		1.00000	0.2	1.0	3.7	10.0	100.5	1.0	2.0
							100.0	99.3	1.4	1.3
		y = 1200.4x − 3213.9					10.0	102.9	4.2	7.3
Aldicarb	10.0–500.0		0.9995	3.0	10.0	−16.1	100.0	99.6	1.7	0.9
							500.0	100.6	0.6	0.9
		y = 242.56x − 308.51					10.0	98.6	6.3	14.2
Metolcarb	10.0–1000.0		0.9992	3.0	10.0	9.4	100.0	96.6	3.3	1.2
							500.0	97.9	3.8	2.3
		y = 1524.9x − 598.33					10.0	103.6	3.9	1.4
Propoxur	5.0–500.0		0.9996	2.0	5.0	−6.5	100.0	99.8	2.5	1.5
							500.0	99.8	0.9	1.8
		y = 22,528x + 10,821					1.0	86.5	1.9	4.3
Carbofuran	1.0–200.0		1.0000	0.3	1.0	3.8	10.0	102.9	2.7	2.8
							100.0	101.2	1.7	0.8
		y = 1192.2x − 166.83					10.0	105.7	4.4	6.3
Carbaryl	10.0–500.0		0.9997	3.0	10.0	−7.4	100.0	96.9	3.1	2.4
							500.0	98.2	1.5	3.7
		y = 2748.7x + 6693.7					10.0	101.9	3.9	3.4
Isoprocarb	10.0–1000.0		0.9996	3.0	10.0	−2.2	100.0	101.4	2.3	2.3
							500.0	101.0	2.5	2.1
		y = 3294.2x + 13,084					10.0	114.0	2.1	2.0
Promecarb	2.0–1000.0		0.9997	0.6	2.0	−9.4	100.0	108.5	2.4	7.1
							500.0	102.8	2.1	2.1

**Table 3:** NEO and CBM pesticides in seven FD cabbage samples by the proposed method (X ± SD, *n = 3*).

Compounds	Sample 1 (μg/kg)	Sample 2 (μg/kg)	Sample 3 (μg/kg)	Sample 4 (μg/kg)	Sample 5 (μg/kg)	Sample 6 (μg/kg)	Sample 7 (μg/kg)
Propamocarb	<LOD	<LOD	<LOD	16.7 ± 1.1	15.5 ± 1.2	35.7 ± 2.1	<LOD
Dinotefuran	<LOD	<LOD	<LOD	<LOD	<LOD	<LOD	<LOD
Oxamyl	<LOD	<LOD	<LOD	<LOD	<LOD	<LOD	<LOD
Nitenpyram	<LOD	<LOD	<LOD	<LOD	<LOD	<LOD	<LOD
Cycloxaprid	<LOD	<LOD	<LOD	<LOD	<LOD	<LOD	<LOD
Thiamethoxam	<LOD	<LOD	<LOD	<LOD	<LOD	<LOD	<LOD
Clothianidin	<LOD	<LOD	<LOD	<LOD	<LOD	<LOD	<LOD
Imidacloprid	<LOD	<LOD	<LOD	<LOD	<LOD	<LOD	78.6 ± 6.0
Imidaclothiz	<LOD	<LOD	<LOD	<LOD	<LOD	<LOD	<LOD
Acetamiprid	<LOD	<LOD	<LOD	<LOD	<LOD	<LOD	4.0 ± 0.1
Pirimicarb	<LOD	<LOD	<LOD	<LOD	<LOD	<LOD	<LOD
Thiacloprid	4.5 ± 0.2	5.7 ± 0.2	<LOD	17.1 ± 0.5	5.0 ± 0.3	<LOD	6.9 ± 0.5
Aldicarb	<LOD	<LOD	<LOD	<LOD	<LOD	<LOD	<LOD
Metolcarb	<LOD	<LOD	<LOD	<LOD	<LOD	<LOD	<LOD
Propoxur	<LOD	<LOD	<LOD	<LOD	<LOD	<LOD	<LOD
Carbofuran	<LOD	<LOD	<LOD	<LOD	<LOD	<LOD	<LOD
Carbaryl	<LOD	<LOD	<LOD	<LOD	<LOD	<LOD	<LOD
Isoprocarb	<LOD	<LOD	<LOD	<LOD	<LOD	<LOD	<LOD
Promecarb	<LOD	<LOD	<LOD	<LOD	<LOD	<LOD	<LOD

## Data Availability

Not applicable.

## Supplementary Materials

The following supporting information can be downloaded at: https://www.mdpi.com/article/10.3390/foods12040699/s1, Figure S1: Chemical structures of the target NEO and CBM pesticides; Figure S2: Effect of extraction time on the recovery of NEO and CBM pesticides; Figure S3: Effect of extraction time on the MEs of NEO and CBM pesticides; Figure S4: Matrix profiles in different partitioning salts for cycloxaprid; Figure S5: Matrix profiles in different partitioning salts for imidacloprid; Figure S6: The influence of ammonium acetate on the peak shape of nitenpyram; Figure S7: The influence of the dilution with phase A on the peak shape of propamocarb; Figure S8: Extracted ion chromatogram of NEO and CBM pesticides at LOQs concentration; Figure S9: The stability of NEO and CBM pesticides in room temperature storage for one day; Figure S10: The stability of NEO and CBM pesticides in 4°C storage; Table S1: Current maximum residue limits (MRLs) for NEO and CBM pesticides in fresh cabbage (mg/kg); Table S2: Normalized signal intensity measured before and after use of 5 mM ammonium acetate. Table S3: Comparison of different methods for the analysis of NEO and CBM pesticides in fresh cabbage.
