# Supplementary material for: Simultaneous Determination of Neonicotinoid and Carbamate Pesticides in Freeze-Dried Cabbage by Modified QuEChERS and Ultra-Performance Liquid Chromatography–Tandem Mass Spectrometry
Source: Foods. 2023 Feb 6;12(4):699. doi: 10.3390/foods12040699 (PMC9955598; doi:10.3390/foods12040699)
Supplement: Supplementary file 1 [file foods-12-00699-s001.zip › foods-2165635-supplementary.pdf]

Simultaneous Determination of Neonicotinoid and Carbamate Pesticides in Freeze-Dried Cabbage by Modified QuEChERS and Ultra-Performance Liquid Chromatography–Tandem Mass Spectrometry

Bingxin Yang [1,2], Sheng Wang [1], Wen Ma [3], Guanlin Li [4], Mengling Tu [1], Zhiyong Ma [2,*], Qinghe Zhang [1], Hongmei Li [1] and Xianjiang Li [1,*]

[1] Key Laboratory of Chemical Metrology and Applications on Nutrition and Health for State Market Regulation, Division of Metrology in Chemistry, National Institute of Metrology, Beijing 100029, China

[2] Beijing Advanced Innovation Center for Soft Matter Science and Engineering, State Key Laboratory of Organic-Inorganic Composites, College of Chemical Engineering, Beijing University of Chemical Technology, Beijing 10029, China

[3] State Key Laboratory of Natural and Biomimetic Drugs, School of Pharmaceutical Sciences, Peking University, Beijing 100191, China

[4] College of Plant Protection, Shandong Agricultural University, Tai'an 271018, China

* Correspondence: mazhy@mail.buct.edu.cn (Z.M.); lixianjiang@nim.ac.cn (X.L.)

Table S1 Current Maximum Residue Limits (MRLs) for NEO and CBM pesticides in fresh cabbage (mg/kg)

Analytes	European Union	China	the United State of America
Propamocarb	20	10	15
Dinotefuran	0.01*	2	15
Oxamyl	0.01	/	0.1
Nitenpyram	0.01*	0.2	/
Cycloxaprid	0.01*	/	/
Thiamethoxam	0.02	3.0	4.5
Clothianidin	0.01*	2.0	1.9
Imidacloprid	0.01	0.2	3.5
Imidaclothiz	0.01*	/	/
Acetamiprid	0.01	1.0	15
Pirimicarb	0.5	1.0	/
Thiacloprid	1.0	/	/
Aldicarb	0.02	0.03	/
Metolcarb	0.01*	/	/
Propoxur	0.05	/	/
Carbofuran	0.002	0.02	/
Carbaryl	0.01	5.0	21
Isoprocarb	0.01*	/	/
Promecarb	0.01*	/	/

* General MRLs of 0.01 mg/kg due to absence of a specific MRLs for the substance in cabbage

Table S2 Normalized signal intensity measured before and after use of 5 mM ammonium acetate

Analytes	Normalized signal intensity (0.1% formic acid)	Normalized signal intensity (0.1% formic acid+5 mM ammonium formate)
Propamocarb	1.0	1.8
Dinotefuran	1.0	5.3
Oxamyl	1.0	0.2
Nitenpyram	1.0	4.8
Cycloxaprid	1.0	1.5
Thiamethoxam	1.0	2.9
Clothianidin	1.0	2.0
Imidacloprid	1.0	1.2
Imidaclothiz	1.0	1.5
Acetamiprid	1.0	1.6
Pirimicarb	1.0	5.0
Thiacloprid	1.0	1.2
Aldicarb	1.0	0.3
Metolcarb	1.0	1.4
Propoxur	1.0	4.4
Carbofuran	1.0	2.5
Carbaryl	1.0	12.2
Isoprocarb	1.0	4.9
Promecarb	1.0	6.3

Table S3 Comparison of different methods for the analysis of NEO and CBM pesticides in fresh cabbage.

Analytical method	Analytes	LOQs (μg/kg)	Detected pesticides in real samples	Ref.
QuEChERS-UPLC-MS/MS	8 NEOs	0.5-1.0	Nitenpyram, thiamethoxam, clothianidin, imidacloprid, acetamiprid, thiacloprid	7
MSPE-HPLC-DAD	179 pesticides	1.2-16.7	Pirimicarb, imidacloprid	8
Electrochemical paper-based device	Carbofuran, carbaryl	\	Carbofuran, carbaryl	38
QuEChERS-UPLC-MS/MS	Isoprocarb, carbofuran, imidacloprid, acetamiprid	0.006-0.02	Imidacloprid, acetamiprid	39
QuEChERS-UPLC-MS/MS	Dinotefuran, thiamethoxam, clothianidin, thiacloprid	0.71-2.26	<LODs	40
QuEChERS-UPLC-MS/MS	9 NEOs and 10 CBMs	1.0-10.0	Thiacloprid, propamocarb, imidacloprid, acetamiprid	In this work

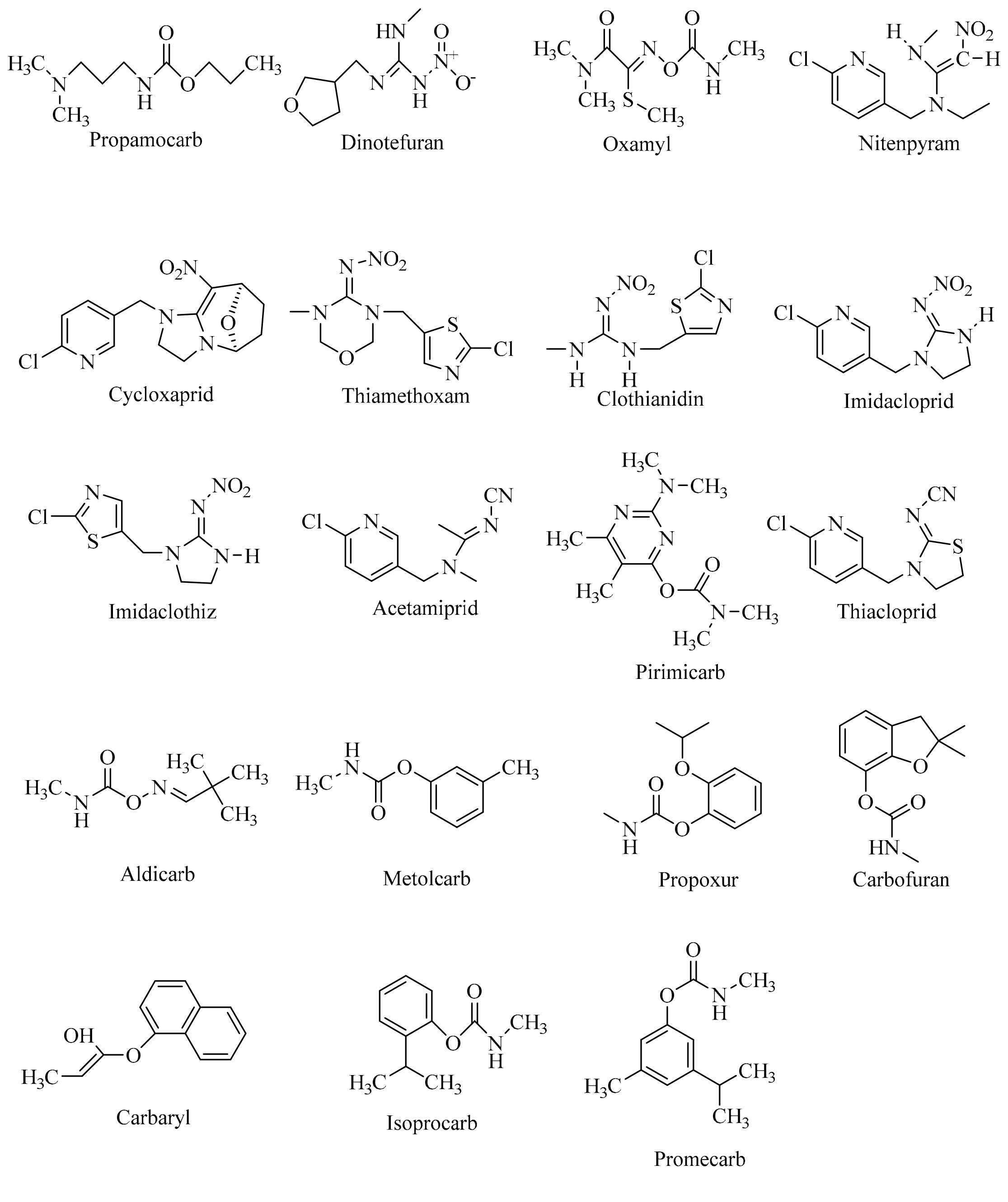

Figure S1 Chemical structures of the target NEO and CBM pesticides

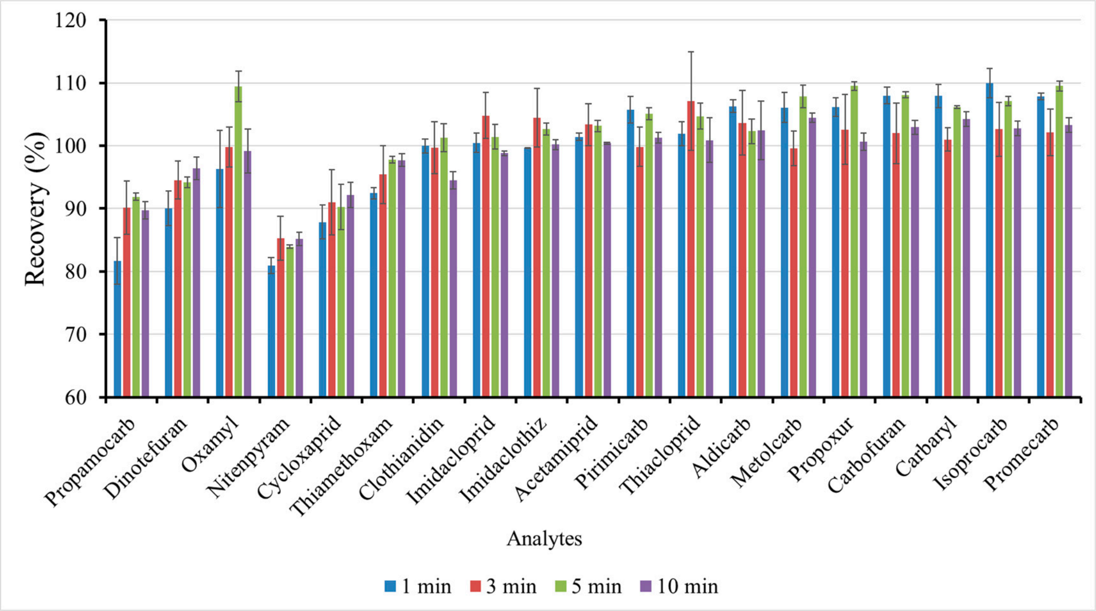

Figure S2 Effect of extraction time on the recovery of NEO and CBM pesticides

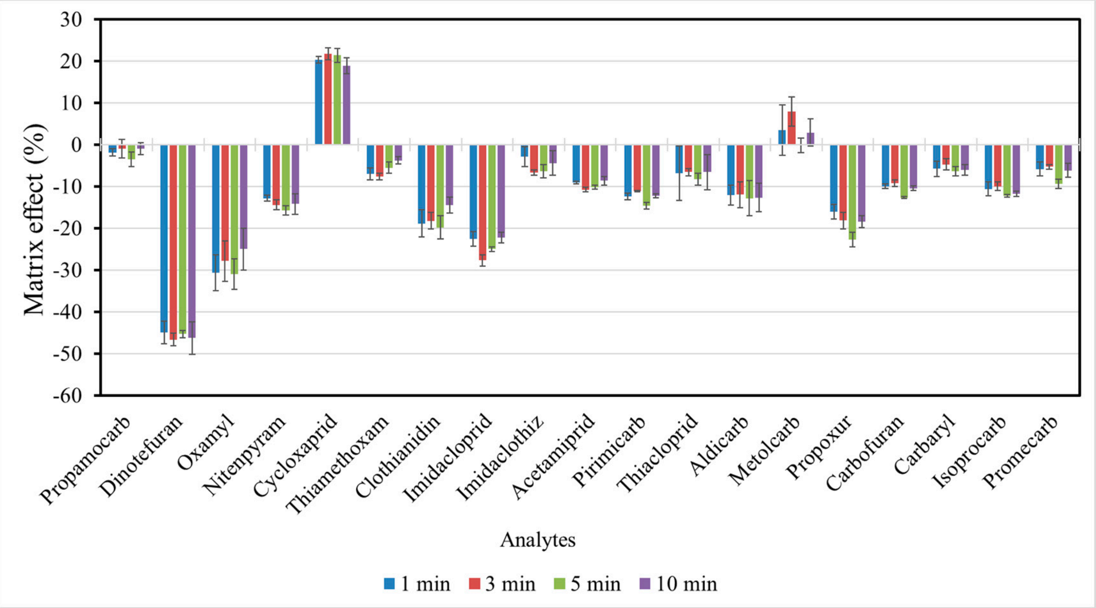

Figure S3 Effect of extraction time on the MEs of NEO and CBM pesticides

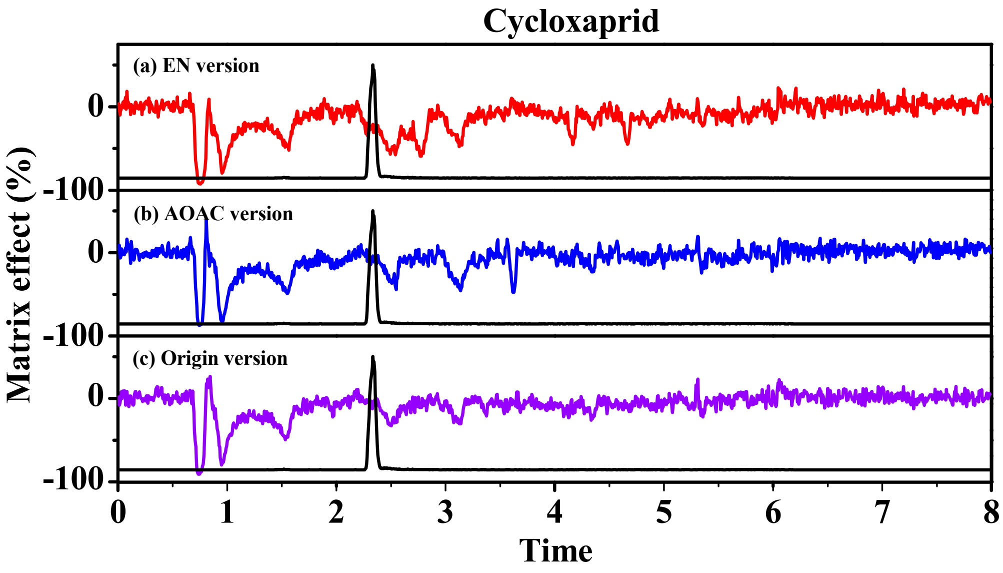

Figure S4 Matrix profiles in different partitioning salts for cycloxaprid

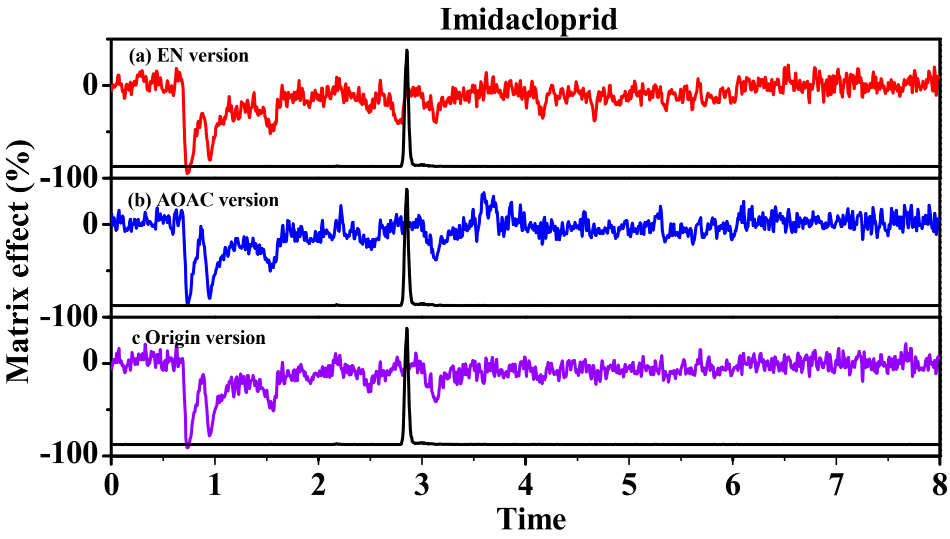

Figure S5 matrix profiles in different partitioning salts for imidacloprid

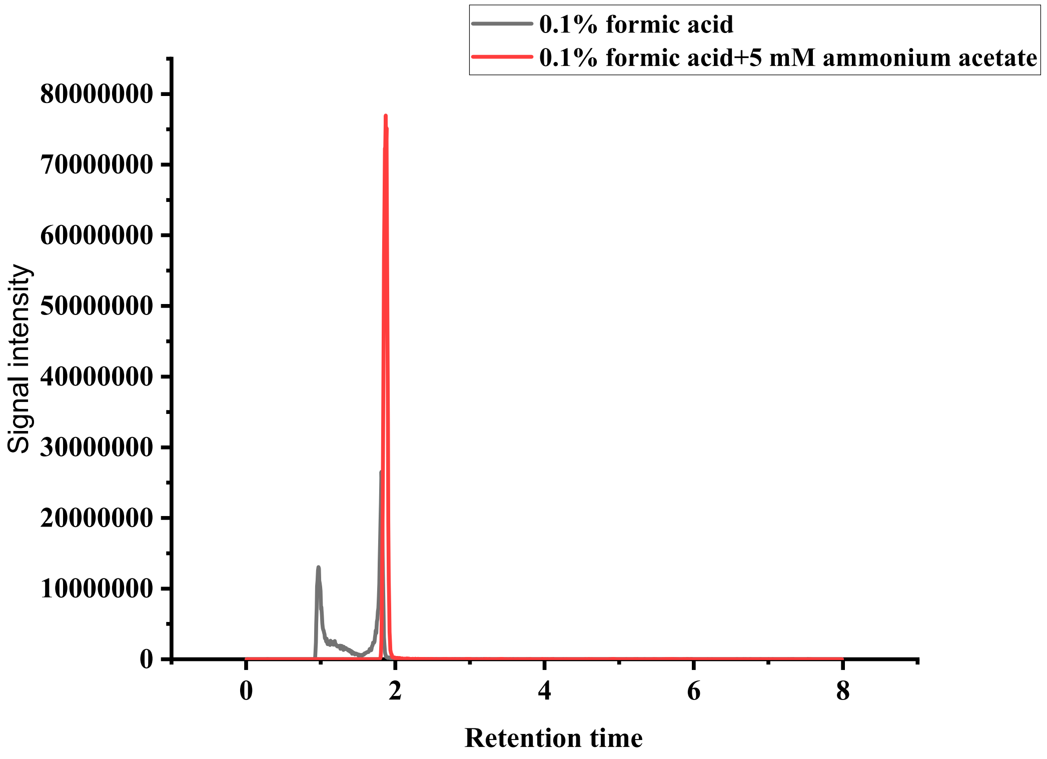

Figure S6 The influence of the ammonium acetate on the peak shape of nitenpyram

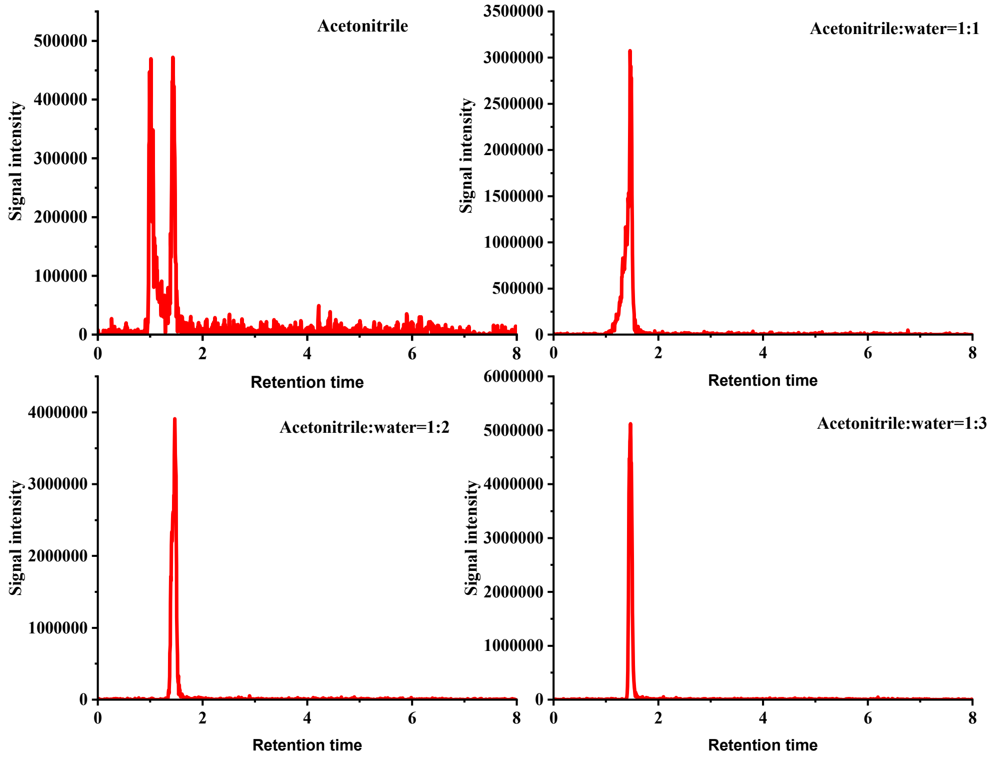

Figure S7 The influence of the dilution with phase A on the peak shape of propamocarb

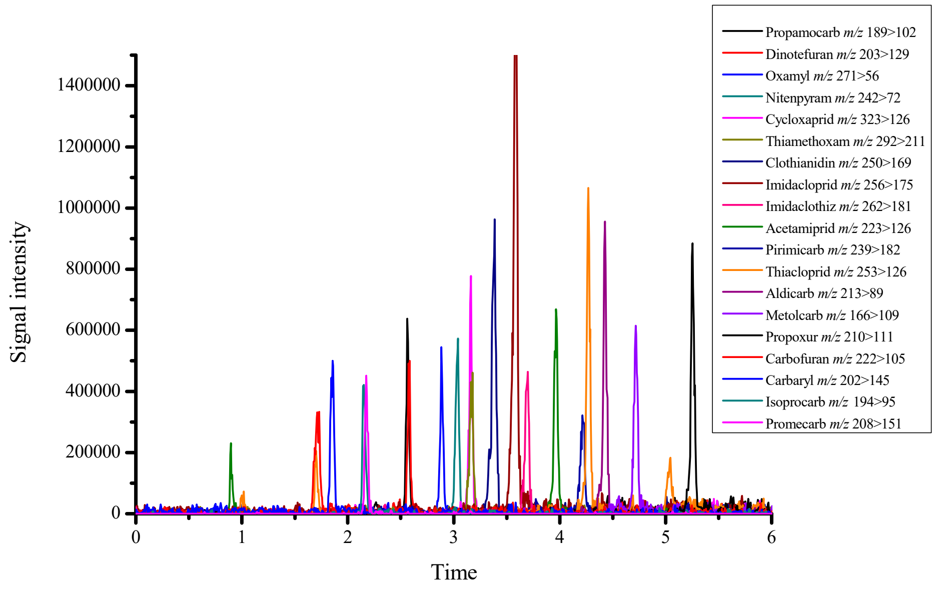

Figure S8 Extracted ion chromatogram of NEO and CBM pesticides at LOQs concentration

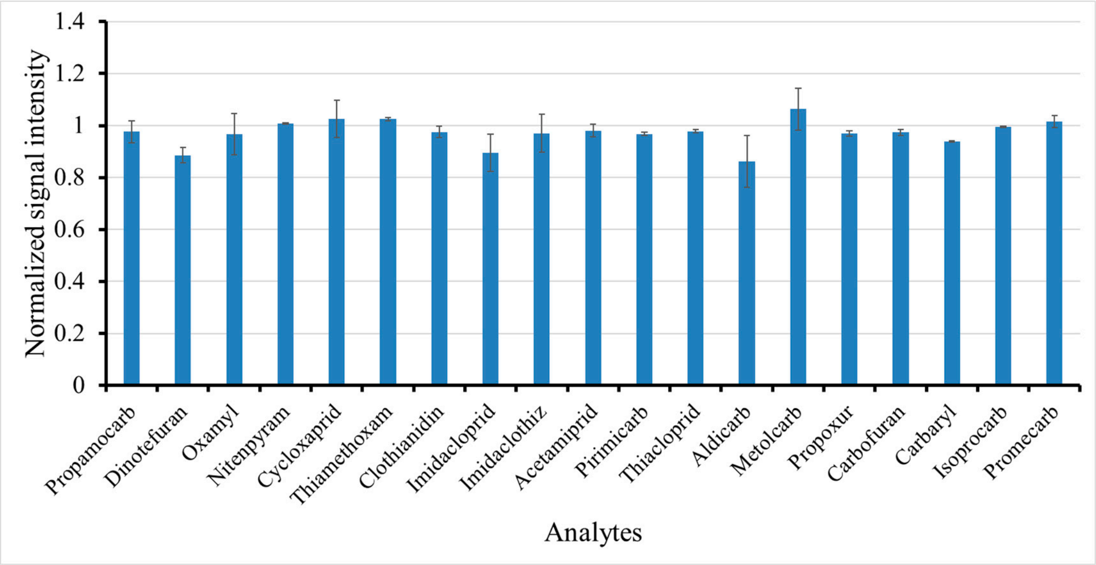

Figure S9 the stability of NEO and CBM pesticides in room temperature storage for one day

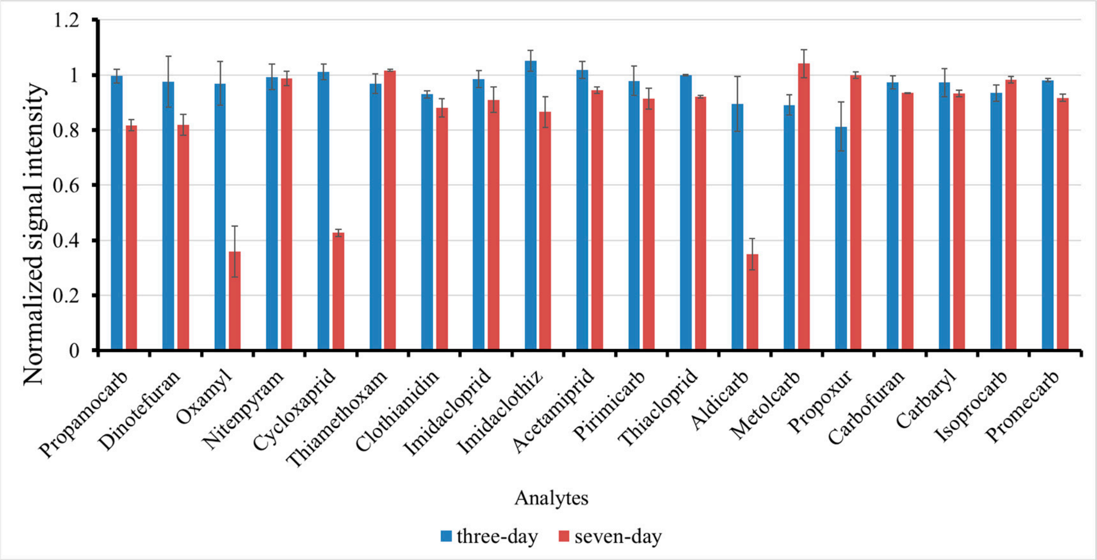

Figure S10 the stability of NEO and CBM pesticides in 4°C storage
